# Coordination processes in partnerships between German governmental organizations and German sports federations in jointly implemented SDP projects

**DOI:** 10.3389/fspor.2022.989284

**Published:** 2022-11-03

**Authors:** Laura Schreiner, Valerie Kastrup, Jochen Mayer

**Affiliations:** ^1^Department of Sports Science, Bielefeld University, Bielefeld, Germany; ^2^Department of Sports and Exercise, University of Education Schwäbisch Gmünd, Schwäbisch Gmünd, Germany

**Keywords:** sport for development and peace, national development policy, systems theory, inter-organizational relationships, coordination processes in partnerships

## Abstract

Sports and physical activity are increasingly used as instruments of development policy. Within this field of sport for development and peace (SDP), one can observe a large number of partnerships between national governmental organizations and national sport federations. These are particularly important although these organizations pursue different core objectives with their SDP engagement. The aim of this qualitative study is to analyze how German governmental organizations and sports federations coordinate jointly managed SDP projects. Against the backdrop of systems theory, we conducted interviews with participants from relevant organizations. The qualitative content analysis revealed that inter-organizational coordination takes place partly in hierarchical and partly in network-based structures. The results show complex patterns between the participating organizations which gain varying degrees of influence on the SDP projects and seek to get specific resources through the cooperations. These should be taken into consideration when designing SDP projects to build stable cooperations that bring all the participating organizations an added value.

## Introduction

In the field of Sport for Development and Peace (SDP), many projects are conducted jointly by different organizations. Consequently, the SDP field is complex and populated by numerous organizations, partnership associations, and networks (1). Partnerships are usually viewed by organizations participating in the SDP field as a way to reach policy goals effectively but can vary in terms of their specific design and objectives (2).

In general, partnerships are viewed as crucial to the SDP field (3, 4). Particularly influential in the field are cooperations between sports organizations and national governmental organizations, which frequently implement joint national SDP projects. However, sports and governmental organizations pursue different organizational core objectives. While governmental organizations promote a development policy under the banner of the Sustainable Development Goals and thus pursue development policy goals such as the fight against hunger and poverty, sports organizations are primarily focused on sport-specific goals (5). From this perspective, the question arises as to how sports and governmental organizations manage to implement and coordinate joint SDP projects.

Although a large body of literature on partnerships between national governmental and sports organizations exists, our understanding of partnerships between national governmental and sports organizations in the SDP field is still limited. The present paper addresses this issue and seeks to ascertain how governmental and sports organizations exactly coordinate joint projects and how both of them ensure that their own interests are considered.

The current state of research identifies a wide variety of reasons and goals for organizations entering into partnerships (2, 6, 7). Governmental organizations have a wide range of goals for engaging in the SDP field, including establishing institutional networks or enabling the exchange of knowledge, for example by providing relevant information, naming contact persons and organizing conferences, forums and symposia on SDP (8). Additionally, governmental organizations are attested to try to gain influence and exert so-called “soft power”, a specific form of power, which, for example, does not require any military means. Giulianotti et al. observe that “Nordic countries tend to fund programs while acting as pro-development ‘regimes of goodness’; other nations operate more instrumentally within Sportland^1^ to gain greater influence within local sport federations” (7). Thus, entering into partnerships may well be aimed at gaining influence over the partners. Giulianotti et al., therefore, seek to ascertain the extent to which nation-states are primarily concerned with exercising “soft power” in their SDP engagement (7). This is also the focus of Garamvölgyi et al., who describe how, through sport and soft power, a positive image of the nation can easily be promoted abroad (9).

Sports organizations, in turn, have other goals pursued in SDP engagement (5) such as social engagement, new income and/or new markets, talent scouting, marketing-specific goals, or increasing the sport's participation (10, 11). Additionally, AlKhalifa and Collison identify–generally spoken–“increasing the amount of activities to raise awareness of development goals,” “promoting the organization by improving the track record,” and “expanding networks,” *inter alia*, as pursued goals (6). However, one can observe that the respective goals are not openly communicated in every case but in some cases, they seem to be adopted for the relevant audience (11). Furthermore, Peachey et al. observe that the goals and motives of organizations working in the SDP field and engaging in partnerships can change, for example, if the goals evolve over time or need to be changed for the partner's satisfaction (12). The latter could be found in their study, especially in funding partnerships (12).

Consequently, SDP projects implemented jointly by sports and national governmental organizations bring together organizations that pursue different goals and purposes. Relatedly, it can be seen that they have different expectations from and a specific influence on SDP projects (5). Hence, a fundamental problem arises: if the organizations have different goals for an SDP engagement, how do they manage to design and coordinate joint projects and to define the goals, content, and tasks of the jointly implemented SDP projects? The subsequent processes of the conceptualization and implementation of joint SDP projects seem to be highly complex and susceptible to disruption. Lindsey and Banda find that there are partnerships in which power is distributed equally among the participants, as well as others with unequal power relations (2). Giulianotti observes that collaborative projects between what he calls “strategic developmentalists,” who “tend to pursue strategic developmentalist SDP policies” (8) and among which he includes national governmental agencies, intergovernmental organizations and sports federations are “characterized by top-down management and network-building techniques for knowledge transfer across the SDP sector” (8). Governmental organizations typically prefer large organizations with connections and influence (13) and for the most part, SDP projects are strongly marked by government-oriented goals since SDP agencies frequently adopt objectives set down by (inter)national government bodies. At the implementation level, on the other hand, government organizations often copy the methods and practices of NGOs and sports organizations (5, 8), for example, by using prominent athletes as ambassadors of specific campaigns or by “defining their own SDP work as ‘corporate social responsibility”’ (8). Consequently, non-profit organizations are frequently influenced by inter-organizational interactions, especially those in turn leading to a strong pressure on them to adapt (12, 14, 15). Due to the increased involvement of government organizations, NGOs primarily concerned with the use of SDP, in particular, are increasingly influenced by state logic. They have to adapt to a wide variety of institutional logic, especially if they rely on funding to secure the support of the participating organizations (16). However, not all organizations are equally affected by this pressure to adapt (12), and Svensson and Seifried suggest that SDP-oriented organizations are significantly more affected by this than regular sports organizations are (16).

Overall, research on organizational processes shows that the conceptualization of partnerships often requires a highly complex process in which one partner (mostly governmental organizations) can often exert more influence than others. The latter is therefore strongly pressurized to adapt. However, it is not clear yet how governmental and sports organizations exactly coordinate the joint projects, considering their specific interests.

Additionally, it appears that the dominant actors in the SDP field are mostly organizations from the Global North, which leaves little opportunity for organizations and actors from the Global South to exert influence (2). They often bring a lot of resources to the projects and can thus gain interpretive authority over the project goals and content. Nicholls et al. even observe that the Global North can often decide what is to be implemented in the SDP projects and what is recognized as proof of the projects' success. Colonial ideas are thus perpetuated (17) and the dominance of the Global North and its own perspective are constantly reproduced. Accordingly, SDP projects are often characterized by “donor-driven priorities and top-down approaches” (17). The local level as well as the target groups, however, are mostly not even involved in the design of the projects [see, for example, Hayhurst (18)].

Partnerships between the organizations of the Global North are thus likely to be highly influential, but it is precisely these partnerships that have so far received little attention in research. This is why, in this article, the question of how joint SDP projects between governmental and sports organizations are coordinated, will be addressed using the case of Germany. This is a good example for us to study partnerships between national governmental and sports organizations.

Against this background, this paper addresses the question of how German national governmental and sports organizations coordinate jointly managed SDP projects. It is also important to analyze how the goals and contents of the projects are determined and how the organizations ensure that the projects meet their specific interests. Thus, the goal here is to paint a differentiated picture of how the inter-organizational relationships and coordination processes between sports and governmental organizations are formed in joint SDP projects. In the following pages, the theoretical background for analyzing partnerships between German governmental organizations and German sports federations in jointly implemented SDP projects is discussed, referring to Niklas Luhmann's systems theory (19). The central research questions for our qualitative study are presented on this basis.

## Inter-organizational relationships and coordination processes from a systems theoretical perspective

In order to cast light on the inter-organizational relations between sports and governmental organizations, we chose Luhmann's (19) systems theory and its application to the sports context [e.g., (20)] as the basis for our theoretical considerations. This theoretical approach has only recently been used in SDP research (5). First of all, this approach adds an additional and comprehensive theoretical perspective within a still “undertheorized” (21) field. Second, Luhmann's systems theory allows focus on social structures in general and organizational structures in particular (22). Moreover, it conceptualizes the organizations involved as specific social systems that are fundamentally oriented to the logic of their respective societal system. Additionally, the approach allows the identification of different resources the organizations can provide. Finally, it allows observation and analysis of interorganizational relations to shed light on the question of how organizations coordinate joint SDP processes and how they can influence other organizations. In the following we will, firstly, present relevant basic system theoretical assumptions. Based on these, secondly, we will show in which way the organizations can potentially exert influence on the cooperations and, thirdly, the form in which the coordination processes can take place.

If we understand modern western society in Luhmann's (23) sense, sport and governmental organizations belong to different functional subsystems, namely, the sports system and the political system. Like all functional subsystems, both are autopoietic, self-referential and self-contained, acting according to a system-specific code and oriented to a specific function (24–27). From this point of view, the sports system consists of “all actions (.) whose purpose is the communication of physical performance” (28) while the political system has the function to hold the capacity for collectively binding decisions (29).

In order to fulfill the system-specific purpose and function, it “requires appropriate material as well as immaterial resources” (30). Among these, according to Miller, are “material and instrumental resources” (e.g., money, commodities, material goods, ideas, and knowledge), “social resources” (e.g., contacts, networks, trustworthiness and respect), and “cultural resources” (e.g., experience, traditions and values) (30). In order for the sports and the political systems to be able to fulfill their respective functions, they require specific resources on the one hand but are also able, on the other hand, to provide other systems with these in different measures.

Functional subsystems act autonomously but they can render services to other subsystems (31). However, no direct access is possible from the system to its environment and vice versa. Information which reaches the system from the outside can only influence internal system processes when it is considered as relevant and is then integrated into the system's own structure (32). Hence, in modern western societies the sports system can render services to the political system, for example, by making a suitable instrument available for development policy but the political system can never gain direct access to the sports system. This can only be done by the sports system itself and it will only do this when the information received is considered relevant.

When an organization attempts to influence this process of another system's self-determination in a targeted way, it is known as “governing.” This means that the organization tries to intervene in the other's communication system (33) so that the latter organization governs itself in the intended direction as autonomously as possible. The organization to be governed can then “react as desired, or differently, or not at all” (34). If the political system thus wishes to prompt the sports system to make changes to its own system structures, it cannot intrude directly on the sports system. It can, however, attempt to get the sports system to modify its own structures itself. Ultimately, though, this always remains a mere attempt because it is not certain how the sports system will react to the information from outside. In order to solve this problem and facilitate collective action with the common objectives, the so-called governing instruments such as money, power, sympathy, trust or knowledge may be put into effect (35). These–symbolically condensed–communication instruments enable a contextual governing where the governing organization has no need to intervene in the internal structure of the organization being governed. Rather, this latter organization can be induced to govern itself by offering certain incentives (36). The political organization might offer money, for example, in order to induce the sport organizations to undertake collaborative projects and render certain services. However, sports organizations will only reflect on this prospective collaboration if the opportunities related to it are, against the backdrop of the system-specific code, able to awaken interest within the system and consequently promise further gain. In case a collaborative project is agreed to, inter-organizational cooperation can be coordinated through three different ways: (i) Hierarchical coordination, (ii) democratic (or market-oriented) coordination, and (iii) network- (or negotiation-) oriented coordination (37). Under hierarchical conditions, the delegation simply takes place from top to bottom (37). The second form of coordination is the direct antithesis; in democratic and market-oriented coordination processes, a prompt decision can be made based on supply and demand–with the proviso that, on account of price mechanisms, the conditions of free access and withdrawal are met (36). The third form, network-oriented coordination, is located between the two preceding ones (38). Here, it is a question of finding ways and means which are acceptable to both the sides; it offers two systems of equal rank the opportunity of preserving their autonomy and reaching joint solutions (37).

**Figure 1:**
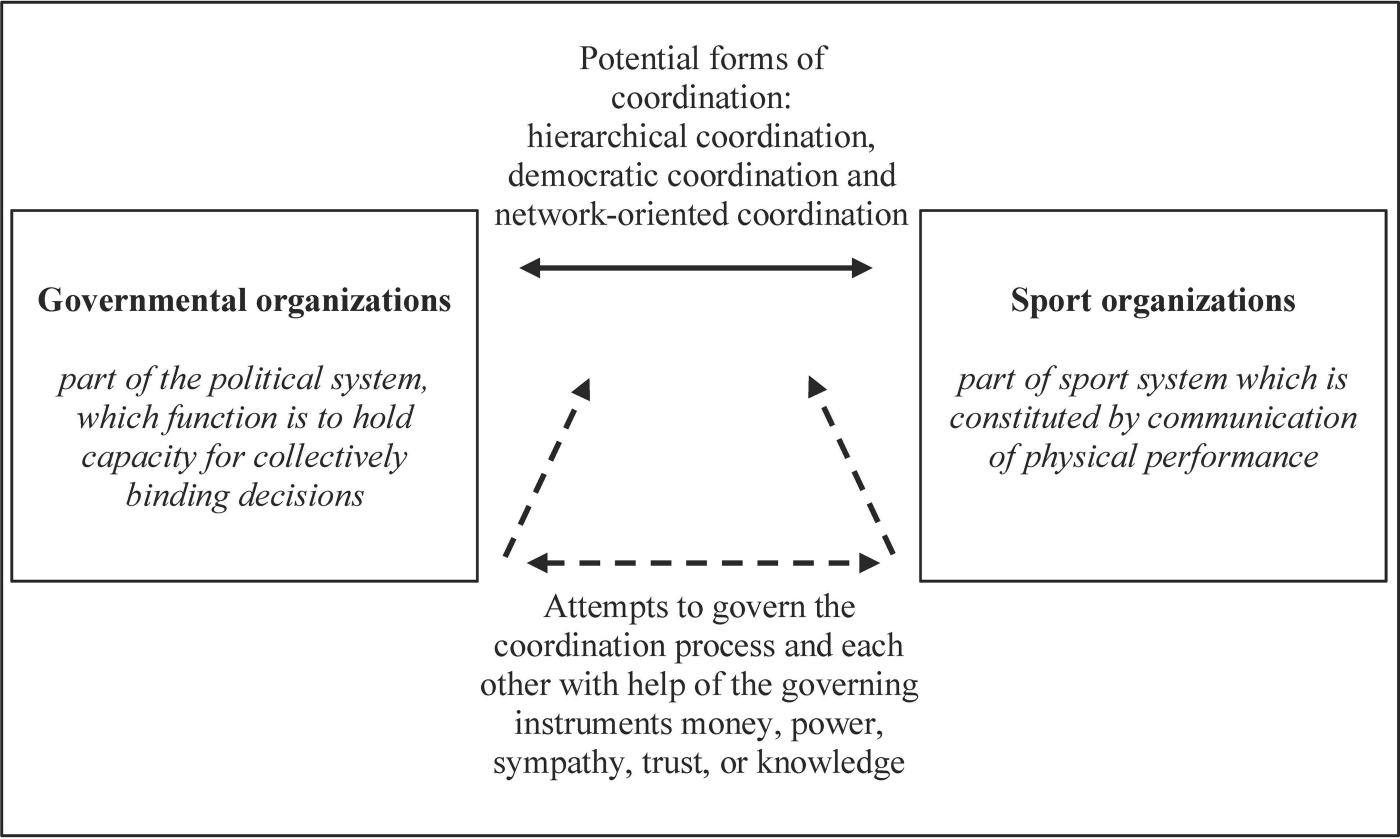
Potential forms of the collaboration's coordination and possible governing instruments.

In summary, as Figure 1 illustrates, from a systems theoretical perspective, sports and governmental organizations operate in different systematic contexts and pursue their own specific goals. Their collaboration can be coordinated in different ways and the organizations can try to govern the collaboration or each other with the help of different governing instruments. The forms of coordination processes ultimately used in inter-organizational cooperation within SDP projects are very much dependent on the existing organizational structures and the goals of the individual stakeholders as well as on the applicable legal framework and on the particular national characteristics. Against the background of this research, it is to be expected that the collaborative projects are managed top-down and marked by government-oriented goals since SDP agencies frequently adopt objectives set down by (inter)national government bodies. In their practical implementation, however, they are likely to bear a strong imprint of the sports organizations. Thus, within a systems theory context, the organizations will at no time ‘forget’ their own systemic context and dedicate themselves without hesitation to other goals. Rather, the projects and programs must continue to serve their own organization-specific goals. Consequently, organizations will only invest their resources when their own goals are put into effect. Therefore, it can be assumed that the necessary coordination between sports and political organizations is predominantly carried out by means of hierarchical and network-oriented coordination processes.

Building on the considerations presented so far, the following research questions are to be answered based on a qualitative study referring to Germany's SDP projects.

(1) What aims are pursued by the sport and governmental organizations and what resources do they expect to receive when collaborating to carry out joint SDP projects?
(2) What role do governing instruments such as money, power, love, trust, or knowledge play in the collaboration?
(3) To what extent are the forms of coordination hierarchical, market- and negotiation-oriented?

## Materials and methods

In order to reconstruct and analyze the coordination processes between governmental and sport organizations, we conducted a qualitative study referring to German SDP projects and German organizations. Within this qualitative research approach (39, 40), guided interviews with participants from relevant German organizations were conducted.

### The case of German SDP projects

Germany was chosen because its SDP policy is characterized by its close cooperation with sports federations. This may be explained not the least by the current organization of the field of sports as well as by the history of organized sports in Germany which “illustrates how closely the national organization of sport is connected to the political sphere” (34). Not only on account of this close cooperation between government organizations and sports federations in the area of SDP but also on account of the general characteristics of its organization of sport, Germany is a characteristic example for many other European sports systems. Especially in the countries of northern and western Europe, sport is organized to a large extent, as in Germany, in voluntary non-profit sports clubs (34, 41). This means that most of them are not profit-oriented but pursue charitable goals. In the case of German sports clubs and associations, these goals are mostly related to promoting either sport in general or certain types of sport (34). Furthermore, “volunteering of the members [is] a basic characteristic of sport clubs all over Europe” (41). Against this backdrop Germany, on account of its structures and networking arrangements encompassing sport and politics, is a good example for other countries with which to illustrate the coordination processes in collaborative ventures between sports organizations and political partners.

### Research approach and data collection

The German governmental organization, which administers German development policy, is the Federal Ministry for Economic Cooperation and Development [Bundesministerium für wirtschaftliche Zusammenarbeit und Entwicklung, BMZ; (42)]. For technical cooperation, the BMZ primary collaborates with the German Society for International Cooperation (*Deutsche Gesellschaft für Internationale Zusammenarbeit*, GIZ) which is an independent organization but wholly belongs to the German federal state (43). Since the GIZ is in many cases responsible for putting into practice the BMZ's development policy projects, and the SDP projects as well, it is also included in the analysis.

**Figure 2:**
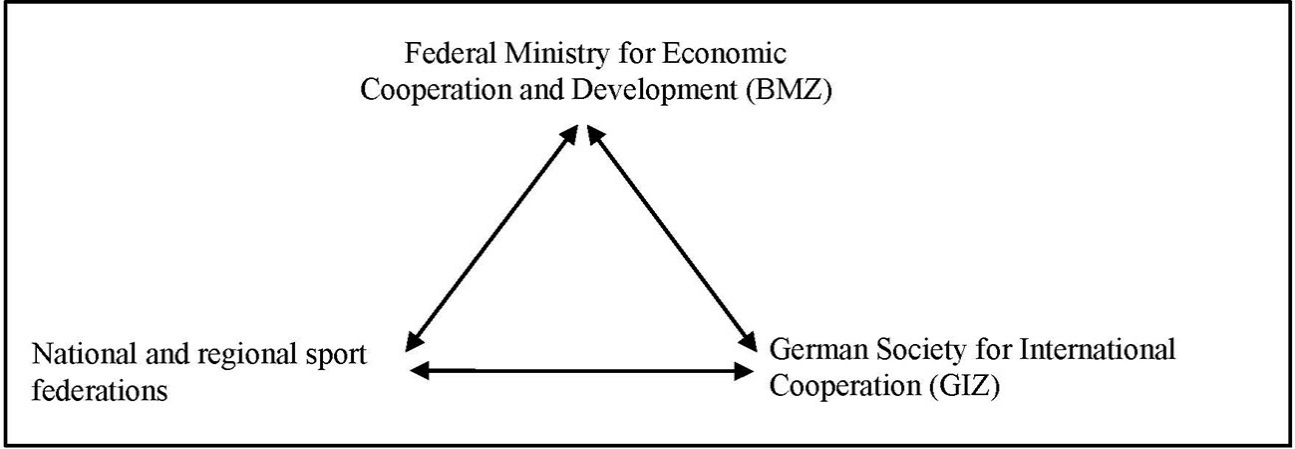
Organizations included in the analysis.

Building on this, we investigated the sports organizations with which the BMZ and the GIZ frequently cooperate within the framework of SDP projects. By means of searches on the appropriate websites, these sports federations were identified and interviews were requested. Most of the requested interviews were accepted, although one national sports federation declined, justifying it with a lack of staff capacity. Ultimately, this led to the inclusion of two state bodies (BMZ und GIZ), a national sports confederation and two regional federations (see Figure 2).

In the case of the state bodies, several representatives were consciously chosen to take in the perspectives of both employees working in Germany and abroad. Thus, in total, seven expert interviews (40) could be conducted. This seems to be a small number of interviews. But on the one hand, there is only a small number of sport and governmental organizations cooperating in German SDP projects and on the other hand, most of the sport organizations only have a small staff responsible for the SDP projects. Thus, we have included most of the relevant perspectives.

Before carrying out the semi-structured interviews, guidelines were drawn up consisting of the theory-based questions on the aims of each of the organizations, on the processes of designing and implementing SDP projects, and on their collaborative ventures with the other participating organizations. In this respect, the interview guideline consisted of a brief introduction, followed by a discussion of the role of SDP for the respective organization and the goals to be achieved. This was followed by open-ended questions on the specific design of the SDP projects, the design of the collaboration with other organizations, and the selection criteria for partner organizations, as well as the reasons for the collaboration. Additionally, the interview guideline contained questions about the resources brought in, the distribution of roles and tasks, and the relationship between the organizations and their goals. An attempt was thus made to gain as holistic and viable a picture of the partnerships as possible through not only the complex questions but also the totality of the interviews.

All interviews were conducted (in six cases, in person and in one case, *via* Skype) by the same interviewer between October 2018 and January 2019. The interviews lasted between 45 and 135 minutes; they were recorded by a digital dictation device with the permission of the interviewees, subsequently transcribed, and then evaluated by means of content analysis.

### Data evaluation and interpretation

The interviews were evaluated using Mayring's qualitative content analysis (44), which has now become an established evaluation method (45). With regard to the concept-structuring analysis technique and an object-related procedure, it was stipulated that the analytical unit could range from a single word to up to several connected sentences. The system of basic evaluation categories was established deductively based on the theoretical considerations, leading to the following four main categories:

(1) Organizational aims of engaging in SDP cooperations.
(2) Resources expected to be received in the cooperation by the organizations.
(3) Indication for use of the governing instruments such as money, power, love, trust, or knowledge.
(4) Indication for hierarchical, market- and negotiation-oriented forms of coordination.

During the processing of the material, these main categories were further developed and refined inductively, as proposed by Gläser and Laudel (40). The references were then categorized, the extracted material was paraphrased, sorted, and abstracted, and the errors were eradicated. On this basis, the key results were summarized for each of the theoretical lead questions. These are presented, subsequently interpreted, and discussed in the light of the following preliminary theoretical considerations.

The interview passages presented below are anonymized and attributed to interviewees with the following abbreviations: “Sto” for an interviewee belonging to a government/state organization, “RSpo” for an interviewee belonging to a regional sports federation, and “NSpo” for an interviewee belonging to a national sports federation. Both the organizations and the interview partners are assigned a number (IP1 to IP7).

## Results

### Aims, resources and governing instruments

According the Luhmann's general system theory the participating organizations strive for different aims and resources when collaborating. In doing so, they make use of different governing instruments. As shown in the following, state organizations do seek for sports organizations' networks, reputation, and proficient staff whereas sport organizations are looking for material resources and state organizations as leading authorities.

#### State organizations seeking for sport organizations' networks, reputation, and proficient staff

Looking at the resources that state organizations primarily strive for, it becomes apparent that three different resources are most important: social, material, and instrumental resources. In the following, we will focus on these three types of resources.

##### Social resources

Sports federations contribute a number of social resources which are repeatedly described in the interviews as being of particular importance for the state organizations. The role of the network is described by one interview partner as follows: “Through them [i.e., the sports federations; authors' note] and through their networks, we also get the instructors and the coaches we need for our work” (StO1-2, p. 64f). For example, the national sports federations have pools of possible coaches for work abroad (StO1-7, p. 112); they know “who's suitable” (StO1-7, p. 360) and they have the right to decide on the personnel to send in consultation with the state agencies. Besides instructors and coaches, there is also the question of other experts (NSpo1-3, p. 290ff.) as well as the contact with implementation partners since “all sports organizations have an international network at their disposal” (StO1-7, p. 120). These networks of the sports federations are even cited by IP2 as the reason why sports federations are included as partners in carrying out an assignment (StO1-2, p. 213ff). Hence, networks–as resources contributed by sports federations–appear to be of exceptional importance for the participating state organizations.

In addition, the reputation and name of the sports federations are described as being especially important for state representatives (StO1-2, p. 482ff) because sports federations are recognized abroad (StO1-2, p. 491ff) and so, can make contact with target groups easier–or even possible–for state organizations.

##### Instrumental and material resources

Other resources that are important for state organizations are knowledge and staff.

On the one hand, state organizations need knowledge about sports pedagogy and methodology (NSpo1-3, p. 291) and on the other hand, they require specific knowledge in areas of development cooperation. In more concrete terms, this means that it takes the form of advice (NSpo1-3, p. 274ff.), training (RSpo1-4, p.18) and drawing up materials such as manuals (StO1-2, p. 515), concepts and curricula. In addition, the sports federations provide network-based knowledge by being able to recommend the appropriate partners to state representatives in development cooperation (or also advise against certain potential partners). As a result, knowledge offers the greatest measure of influence for sports federations. In sports related questions they are credited with a great degree of expertise and it is assumed that, when dealing with certain topics, professional competence “quite clearly lies with the sports organizations” (StO1-7, p. 366).

Sporting expertise is described as being absolutely indispensable in SDP projects “because it isn't just a matter of saying “OK, the ball in the middle and off we go.” Quite the opposite: these measures must naturally be well thought out, pedagogically as well as methodologically” (NSpo1-3, p. 294ff.). Going beyond the conceptual field, it is through knowledge that sport organizations can govern, for example, by being able to influence the way in which a building under construction is equipped and used (RSpo1-4, p. 527ff.) or how manuals are drafted (StO1-2, p. 515f.). Furthermore, sport organizations can provide state organizations with employed staff (for example, the federations' own officials) (StO1-7, p. 497ff), coaches sent abroad (RSpo1-4, 171; StO1-7, p. 80ff) and delegation members of scrutiny missions (NSpo1-3, p. 242). Thus, sport organizations can decide on the choice of coaches to be sent abroad since it is assured that they have “the appropriate autonomy to decide on the personnel. Always, of course, in consultation but the sports organizations are the ones that know who comes into question” (StO1-7, p. 358ff). Their sporting expertise as well as the knowledge derived from their networks enables them, in combination with “intensively listening to each other” (RSpo1-4, p. 294f.), to have a direct influence on the decision of which coaches are sent abroad and which cooperation partners are chosen.

#### Sport organizations seeking for material resources and state organizations as leading authorities

From the perspective of the national sports federations, there seems initially to have been neither any immediate interest in nor any great attention for a possible engagement in a development cooperation. Being asked about the initiation of the partnerships between the BMZ, GIZ and the national sport organizations in SDP projects, one interviewee, for example, describes:

“It's not that sports federations themselves had any great interest […] in engaging in a development cooperation perspective. Rather, it was just understood as being part of a partnership and that, really, the initiative comes from state players: the BMZ or, in the case of promotion of international sport, the AA [the German Federal Foreign Office] (StO1-7, p. 202ff).”

In the following, it will be shown that the sport organizations especially gain material and instrumental resources in the partnerships. Building on this, it becomes apparent that it is possible for the state organizations to make use of the governing instruments of power, money, and knowledge.

##### Material and instrumental resources

The GIZ considers its personnel to be “development experts” (StO1-2, p. 297) who are credited with having a great deal of “expertise” (StO2-6, p. 1561). Consequently, sport organizations, firstly, gain specific knowledge on development policy through the cooperations. The knowledge provided is put into SDP projects in a variety of ways, for example, by advising the Indonesian football federation in a session organized by the GIZ on the subject of “Football for Development” (StO1-2, p. 140f). Furthermore, the coaches chosen for assignments abroad were schooled by the GIZ personnel to prepare them for their stay in partner countries. According to IP2, this is especially important when they are deployed in areas of conflict such as the Palestinian territories (StO1-2, p. 337ff). Consequently, it becomes apparent that knowledge is put into effect as a governing instrument since the GIZ, in cooperation with sports federations, contributes the related expertise (i.e. on development policy), develops, and supports the project concept. Thus, the GIZ is able to influence the relevant decisions on it.

Secondly, sport organizations need money and staff. Here, it must be borne in mind that, with its projects, the GIZ always acts on behalf of other organizations (primarily the BMZ) which pay the GIZ for carrying out a project or make funds available for a project. In the cases studied here, this means that BMZ funds, partly directly and partly *via* the GIZ, go into the projects and thus, into the partnerships with sports federations. In this way, the national sports federations are bound by finance contracts (NSpo1-3, p. 289ff). The GIZ makes the appropriate payments in the first instance from its own project budget and then attempts to involve the third parties in the projects (StO1-2, p. 444ff). Only in a few cases had the projects already been initiated at the request of other governments and implemented jointly with these. Of particular importance to the sports federations are the financial resources in the form of the personnel employed:

“And they profit above all, of course, from the fact that the GIZ has a worldwide presence. I mean, we have offices and projects underway in almost all the developing countries–and they just haven't got the resources or the manpower. So, they lend their know-how, so to speak. But if it wasn't for us, they couldn't set up an office out there from Germany or such things” (StO1-2, p. 524ff).

Of significance here then is that state organizations have personnel working for them internationally. This is extremely important for the sports federations, which cannot afford to pay this huge volume of staff themselves, but in this way, still have possible contact persons in case they plan to engage in a region or country or need a (contact) partner. According to IP5, the GIZ is, thus, “an outpost for us” (RSpo2-5, p. 354f.). The GIZ's personnel abroad is then a basic structural precondition for sports federations' ability to act. Besides, in some cases, the GIZ even finances personnel “who are directly integrated in the sports federations as specialists” (RSpo1-4, p. 327f).

As a result, one can observe a clear distribution of roles and hierarchical structures within the network. The BMZ, being the agency responsible for German development policy as well as for the related projects and programs, has the authority and the task of implementing the development policy. Thus, the SDP projects are given political legitimacy and influenced by the state organizations since the possibility of working in a development policy setting or being a development policy stakeholder is reserved for officials of the state. “The BMZ is the government ministry which, so to speak, formulates programs for foreign countries, engages in consultations with partner governments and is a political player” (StO1-7, p. 218ff). However, in doing so, the BMZ does not need to act alone at all levels; rather, it can “mandate the GIZ or […] other implementing organizations, for example the KfW [a German bank which finances development projects around the world; authors' note], in financial cooperation” (StO1-7, p. 220ff). The BMZ thus has the political legitimacy (and the task) of actively engaging in development policy and can confer a mandate on implementing organizations which then carries out the applicable projects and programs. The other way round, the GIZ fulfills the corresponding assignment of the federal ministry to act on its behalf and implements government development cooperation projects and programs. By means of this clear distribution of roles and the hierarchical structures, the BMZ is able to make use of power- and/or money-based governing. For the national sport organizations, it is clear that “after all, the large projects that we implement are projects which are commissioned by the BMZ together with the GIZ” (NSpo1-3, p. 178ff). Consequently, throughout the cooperation, all the participating organizations are aware of who the contracting authority is and who the implementing agency is (StO1-7, p. 217ff). Accordingly, the BMZ can, for example, lay down the geographical priorities i.e., the target countries (NSpo1-3, p. 397f) and the overarching aims (StO1-2, p. 183ff) while the partners put these priorities into practice. In this regard, the state organizations are the leading authorities within the network with the power to steer the direction of the joint SDP projects.

### Forms of coordination

When analyzing how the cooperations are coordinated, we can identify different forms of coordination between the participating organizations.

#### The GIZ in cooperation negotiations with sports federations

The GIZ's cooperation with national sports federations differs from its cooperation with regional sports federations. One reason for this is that both national sports federations, the German Football Association (DFB), and the German Olympic Sports Confederation (DOSB) are officially included as partners in the contract on the BMZ/GIZ sector project “Sport for Development.”

When a new project begins, the procedure is as follows: in most cases, the BMZ–first of all–contacts the GIZ with certain proposals. The GIZ then approaches the national sports federations and together, they make further agreements (NSpo1-3, p. 403ff.). Thus, there is a standard procedure of who takes the initiative. Subsequently, there are so-called scrutiny missions whereby a delegation is set up to inspect the overall conditions on the ground, examine possible project orientations, consult with as many partners as possible and exchange thoughts on the planning (NSpo1-3, p. 241ff.). This means that the “sports federations are already involved in drawing up concepts” (StO1-7, p. 109f.). In the case of the regional sports federations, these consultations take place in a similar form except that as a rule, they are already active on the ground before a collaborative project is initiated. At any point during the cooperation, each organization has a “right of initiative” (RSpo2-5, p. 394), meaning that it can launch an initiative for new suggestions (and propose possible changes). These processes show that, between the GIZ and the national sports federations, coordination is largely based on negotiation. The result of the consultations is essentially a “mutual decision” (NSpo1-3, p. 181) of all the organizations participating although it is also possible that the participants “spent a lot of time discussing whether they can carry on doing things together or not” (RSpo2-5, p. 466f). Thus, organizations do insist on continuing the cooperation unconditionally; rather, they reflect on the relevance of each decision for their own system so that organizations bring along certain requirements and expectations which can then be negotiated accordingly. Consequently, the relationships between the participating organizations appear to be low in conflict.

#### Between hierarchy and negotiation–the BMZ's coordination with sports federations and the GIZ

Coordination between the BMZ and sports federations can be seen to follow a similar pattern to that between the BMZ and the GIZ.

Moreover, in the case of the sports federations, it was shown that at the operative level, the BMZ cooperated with only the national but not the regional federations. Every kind of indirect connection in which, for example, BMZ funds are allotted to regional sports federations is handled through the GIZ.

As the contracting authority for the projects relevant to this paper (NSpo1-3, p. 178ff), the BMZ has the right in many cases to decide what will be implemented in the projects. Coordination, therefore, is marked by hierarchical structures: “What must be said is that the BMZ naturally has geographical priorities. They have target countries in which they are active. That's the political agenda that defines things” (NSpo1-3, p. 397ff). In concrete terms, this right to decide is used, for example, in specifying the target countries and the target issues for which sports is to be used (the latter are, as a rule, determined by the specific priorities in each country). These decisions are mostly based on “political considerations as to why certain things are wanted or why they should happen” (StO2-6, p. 1571f). This means that “the broad frame then is laid down so to speak by the BMZ, i.e., by politics […] or by the government” (StO1-2, p. 541f). Thus, these requirements form, to some extent, a framework within which the organizations operate in the course of the project (StO1-2, p. 541f). However, the way in which this framework is then filled in detail is not specified hierarchically by the BMZ but is merely coordinated [for example in “jours fixes” (StO1-2,540)] with the participating organizations in the form of consultations (StO1-2, p. 540f). Hence, the BMZ “approaches the GIZ with these political requirements” (NSpo1-3, p. 404) and the specific design of the projects–especially in the case of projects abroad–is put in the hands of the GIZ and/or carried out in consultation and negotiations with it.

On the whole, the coordinating structures between the BMZ and the GIZ as well as between the BMZ and sports federations are seen to be both hierarchical and negotiation based. Especially in the areas of political dialogue, strategic (key) decisions, and political questions, the BMZ takes up a clearly dominant position of which, it takes full advantage. However, in areas other than those just listed, the BMZ makes far less use of the hierarchical forms of coordination and, to a large extent, removes itself from the decision-making process either completely (although in many cases keeping itself informed about the decisions made) or negotiates the structures with its partners.

**Figure 3:**
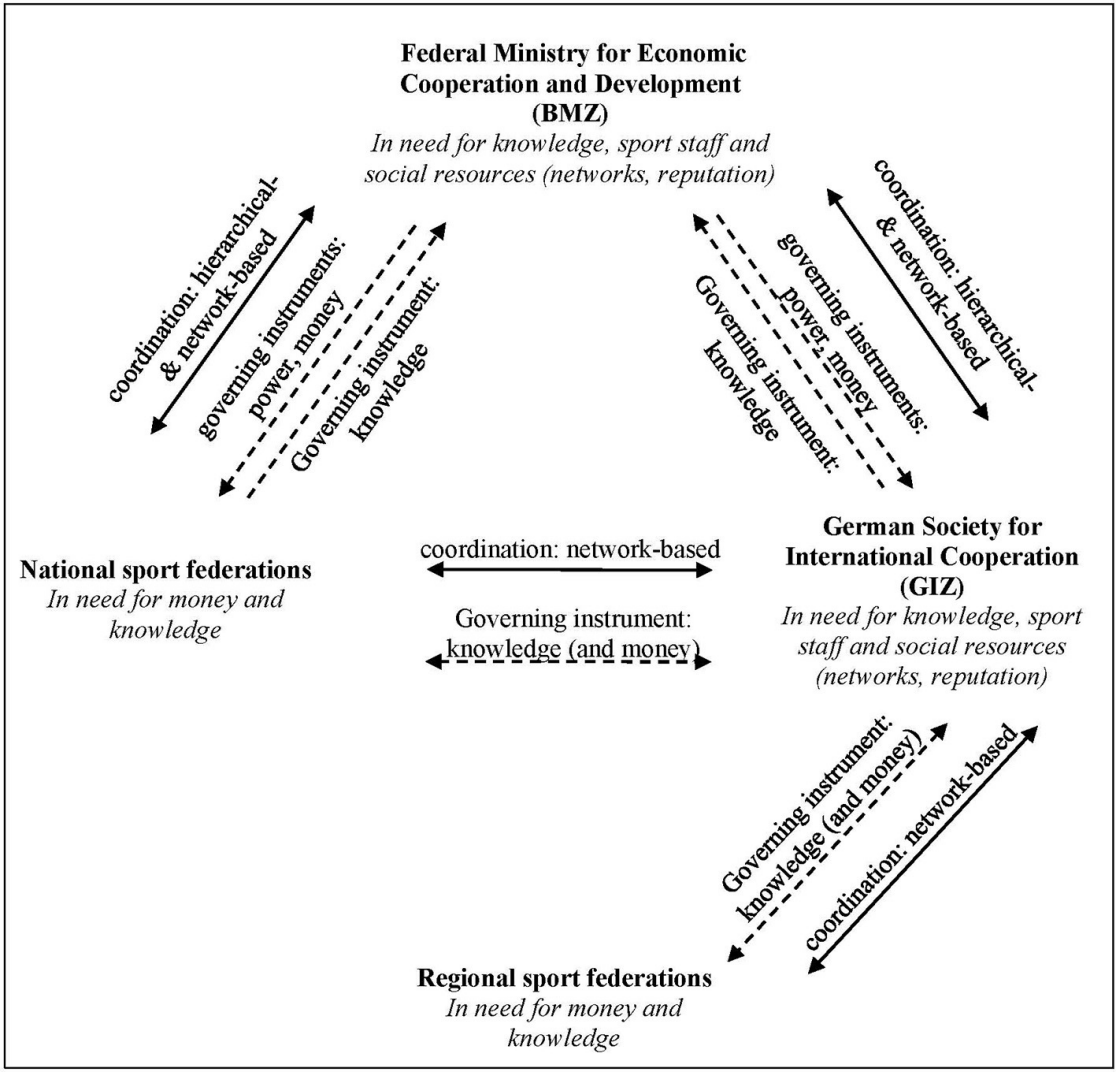
Coordination processes, resources brought in, and governing instruments used.

Based on the preceding theoretical considerations and the findings, Figure 3 summarizes our results. Figure 3 shows a complex pattern of relations between the participating organizations which need a variety of different resources and make use of different governing instruments. The interviews reveal that coordination primarily takes place in hierarchical and network-based forms and the organizations especially use the governing instruments power, money, and knowledge. Particularly striking is that all the organizations require specific knowledge and, therefore, every organization has a potential influence on the joint SDP projects and the other organizations because of the others' knowledge requirements.

## Discussion

The aim of the present study was to venture a look behind the scenes at individual SDP projects and place a focus on the relations between the organizations cooperating in SDP projects. This is intended as a contribution to the ongoing research discourse by attempting to make possible “more integrated and process-oriented understandings of how SDP programs are organized, funded, implemented, and received within networks and relationships” (1). The systems theoretical approach offers various perspectives to observe social structures on different systemic levels such as society, organization, and interaction in society and within the field of sports (20). In our study, we used an organizational theoretical perspective to describe and better understand the interaction of organizations from different societal systems in the field of SDP. Considering this “sub-theory” within the highly differentiated systems-theoretical approach allowed us to systematically analyze the interorganizational relationships between sports and political organizations by focusing on different forms of resources, governing instruments and coordination.

With reference to the theoretical assumptions that coordination processes are essential for working relationships which are neither well-established nor routine (37), it can be seen that the coordination processes in the cases examined above mainly take place in negotiation-based and hierarchical forms (see Figure 3). To be more specific, the GIZ and the sports federations consult each other and come to an agreement on network structures while the coordination processes between the BMZ or the GIZ and the national sports federations take place partly in hierarchical and partly in negotiation-based structures. According to our theoretical considerations, networks do indeed play a special role in the coordination processes. In this way, the BMZ, the GIZ and the participating sports federations can look for solutions that are mutually acceptable. As a result, we would support the observation of Giulianotti et al. that governmental organizations may exert influence on sport organizations (7). However, our findings indicate that it is possible the other way round as well.

Following the theory-driven distinction of different forms of governing instruments, the empirical analysis reveals that sports federations govern, above all, by knowledge while the BMZ attempts instead to manage future communication contingencies by means of power and money–and the GIZ with knowledge and money (see Figure 3). This theory-guided empirical finding also supports Lindsey's and Banda's observation of the different extents of power inequalities in the cooperations of SDP organizations (2). Our results do also reveal that state organizations do exert more power while sports organizations use other governing instruments, especially knowledge.

Considering the needed resources, our results basically support the findings of Giulianotti (8). Moreover, our analysis highlights that sports federations are highly regarded as partners in particular, as they contribute social resources (see Figure 3). We would, therefore, even argue that the required resources are one important reason for organizations to cooperate. Against the systems theoretical background that sports and governmental organizations engaging in SDP do operate within the logic of their own system and pursue different kinds of goals (5), our analysis of SDP projects has shown that the organizations are in need of different resources that the others can provide. Hence, although they may not pursue the same goals, they can provide the required resources to each other, leading to a cooperation becoming possible.

Within our theoretical considerations, we assumed a potential for social conflicts because of the organizations' diverging goals. However, there were no signs of larger “conflict systems” (46), which comprise a communication system of mutually related communication of opposition. Additionally, we could not find any hints of the pressure to adapt on the participating organizations which is in contrast to the findings from Svensson and Seifried about SDP organizations (16). It would be conceivable that the authors are right that the pressure to adapt becomes more apparent for SDP organizations than for sports organizations due to the fact that the latter are significantly less dependent on the state organizations than SDP organizations probably are.

Thus, we argue that both–the lack of conflicts as well as the lack of pressure to adapt–can be traced back to the existing forms of the coordination structures as these primarily take place in network- and hierarchy-oriented forms. In some distinct areas, the organizations accept the dominant position of the BMZ, and in others, they negotiate about the structures of the SDP projects on a participatory basis. As a result, on the one hand, one can observe a clear distribution of roles but on the other hand, both state and sports organizations can flexibly exert influence on the projects.

What is conspicuous when reflecting on these coordination processes is that the target groups themselves do not seem to be involved in any way. Although it might be assumed that consultations with these do take place (in projects other than those considered here), we found no hints of a systematic and formalized involvement. The fact that target groups are not included in the designing of concepts for SDP projects is not uncommon (18) since the projects are still frequently based upon the perceptions of the Global North (47). In this regard, communication theoretical approaches such as our systems theoretical approach appear useful in order to reveal the underlying social structures of communication processes with the target groups. Therefore, it is necessary to focus on the organizational structures, namely the “decision premises” and the “organizational culture” (20) to identify the level of participation and the decision-making authority of the target groups. In this regard, the systems theoretical approach could help to provide new insights into the power relationships between the Global North and the South and its organizations and individual actors.

In addition, further research is needed to show whether the patterns we found for the analyzed cooperations are typical for cooperations between the organizations of the Global North. It is conceivable, for example, that network-based coordination as well as the lack of pressure to adapt are more likely to be found in cooperations between organizations from the Global North.

## Limitations and future directions

The present study takes the example of the German development policy as its subject. The fact that a relatively small number of sports federations participate in most German SDP projects thus resulted in a relatively narrow field of observation where interviews were conducted with only a specific proportion of the participants. Thus, the familiar limitations of qualitative data access apply to this study too. That is to say, the absolute number of those interviewed is rather small, which on the one hand, made it possible to carry out in-depth interviews. On the other hand, though, it did not allow any conclusions to be drawn about the extent to which, for example, decisions are made hierarchically or in negotiations. Consequently, further research projects should include quantitative approaches as well as participatory approaches which are able to give answers to such questions.

At the same time, the sports federations surveyed consisted, to a large extent, of football-oriented clubs. Thus, it is impossible to assess the degree to which the main sport played in a club or federation is reflected in the collaborative projects and whether, for example, very popular sports which have a strong backing in the population (are able to) present themselves in negotiations with greater effect than less known, or marginal, sports and sports organizations.

Since, in the present study, no marginal sports were included, it cannot answer the question of how, and according to which criteria, partners for development policy projects are chosen in the first place. Following Lindsey (13), the question is all the more urgent since it is to be assumed that popular sports and large influential sports federations–on account of the social resources they contribute–are taken into greater consideration than small marginal sports which, within this framework, are thus able to profit less from development policy measures. In this regard, large organizations are expected to have stronger negotiation positions as they do have more relevant resources and (sports) political influence. The duration and depth of the collaboration can also be assumed to play a role because cooperation patterns and areas of responsibility become established over time, and trust is likely to be created. However, if sports organizations do implement all their SDP projects together with governmental organizations, their own activities within the field stand or fall with the cooperation, which might lead to unbalanced power relations and the need to agree or accept the partner's positions.

As a result, in future studies, smaller and lesser-known sport organizations should be included to get to know their expectations and experiences in the SDP field in order to get a better understanding about all kinds of cooperations in the SDP field. For example, it could be found out whether the coordination forms found in the present study typify cooperations between organizations from the Global North or rather between other state organizations and big influential sport organizations.

Besides this, in future research, other (inter-)national examples should be considered, in order to find out whether our results can be applied to other contexts. The German sports system bears characteristics which are to be found in many European sports systems, especially in northern and western European countries. Accordingly, it may be assumed that in many of these countries, similar forms of coordination are predominant. However, in countries where the sports system is organized differently, other patterns of cooperation are to be expected. However, this needs to be further explored. By doing so, more can be learned about cooperations in countries where political or sports systems are organized in a different way than in Germany or rather Europe. Therefore, further studies should take up this issue not only to gain a better overview of international practice but also to make comparisons and perhaps, even present “best practice” examples. The inclusion of other countries also allows to determine the influence of a respective sports discipline on negotiation and cooperation patterns. In Germany, soccer is an extremely popular, marketable, mass media present sport, and it is conceivable that governmental organizations therefore also tend to use this sport for development policy purposes.

Besides this, it may play a role, in whether a particular organization ascribes its SDP engagement to be an important part of its organizational purpose. If the projects are not seen as existential, the potential for conflict is likely to decrease significantly. However, if the projects are of outstanding importance to the organization, the organization is more likely to try to enforce its own ideas and goals with the SDP projects. Hence, conflicts would be more likely to appear.

Our results show complex patterns arising in the analyzed cooperations, but they also bring to mind that every participating organization seeks to gain specific resources in cooperations. This can be particularly helpful for SDP-practitioners when they are trying to identify the right partner for a cooperation.

Considering the required resources, the organizations in question can improve their position in negotiations and within the whole SDP field. Knowledge and communication about the provided, and specifically needed, resources could strengthen the basis for valuable partnerships. This could be especially helpful for smaller organizations with less resources. However, one should keep this in mind: When an organization considers a cooperation–whether with a small or a large organization–it expects to get something from it. And it is precisely this “something” that can be used to strengthen one's own position.

## Data availability statement

The datasets presented in this article are not readily available because the data set includes transcribed interview data. The interview data cannot be made available because anonymization is not completely possible. Requests to access the datasets should be directed to the corresponding author.

## Ethics statement

The studies involving human participants were reviewed and approved by Ethics Committee of Bielefeld University. The patients/participants provided their written informed consent to participate in this study.

## Author contributions

LS was responsible for the literature review, data collection and analysis, and writing of the manuscript draft. VK and JM contributed significantly to the development of the ideas, conceptualization, data collection, and analysis as well as commenting and revising the draft. All authors contributed to the final version and approved the publication of the manuscript.

## Funding

We acknowledge support for the publication costs by the Open Access Publication Fund of Bielefeld University and the Deutsche Forschungsgemeinschaft (DFG).

## Conflict of interest

The authors declare that the research was conducted in the absence of any commercial or financial relationships that could be construed as a potential conflict of interest.

## Publisher's note

All claims expressed in this article are solely those of the authors and do not necessarily represent those of their affiliated organizations, or those of the publisher, the editors and the reviewers. Any product that may be evaluated in this article, or claim that may be made by its manufacturer, is not guaranteed or endorsed by the publisher.

## Footnotes

^1^Sportland is used as a concept to describe SDP “as a distinct field or sector of international development activity.”
